# Low quality evidence supports surgery for gluteal tendon tears, no non-surgical evidence was identified: a systematic review

**DOI:** 10.1186/s12891-026-09519-0

**Published:** 2026-02-03

**Authors:** Trevor Spencer, Anthony Nasser, Nicholas A. T. Brown, Alison Grimaldi, Bill Vicenzino, Paul N Smith, Angela Fearon

**Affiliations:** 1 Discipline of Physiotherapy, Faculty of Health, University of Canberra, Australian Capital Territory, Canberra, Australia; 2 Trauma and Orthopaedic Research Unit, Canberra Hospital, Australian Capital Territory, Canberra, Australia; 3 Australian National University, Australian Capital Territory, Canberra, Australia; 4 Graduate School of Health, University of Technology, Sydney, NSW Australia; 5 Faculty of Health, Queensland University of Technology, Brisbane, QLD Australia; 6 School of Health and Rehabilitation Sciences, University of Queensland, Brisbane, QLD Australia

**Keywords:** Gluteal, Tendon, Hip abductor, Tendon repair

## Abstract

**Background:**

Gluteal tendon tears (GTT) are associated with significant morbidity, similar to end stage, hip osteoarthritis. To date, there is unclear evidence as to what best management for GTT constitutes, and what are the subsequent outcomes for the different management approaches (surgical, non-surgical). The purpose of this systematic review is to document treatments, synthesise evidence, and assess the quality of available evidence regarding the treatments and outcomes associated with symptomatic GTT.

**Methods:**

A systematic review was conducted according to PRISMA (Preferred Reporting Items for Systematic Reviews and Meta-Analyses) guidelines. Six databases were searched, from inception to November 2024 (Cinahl, Scopus, Web of Science, Medline, ClinicalTrial.gov, Cochrane). Inclusion criteria were any research on the management of GTT, exclusion criteria were any other intra-articular pathology. The primary outcome measures were pre-and post-intervention measures for Quality of Life (QoL)(iHOT-12/33) and pain severity (visual analogue scale (VAS)) and were compared to minimally clinically important change. Joanna Briggs Institute (JBI) critical appraisal tool was used to assess methodological quality and Grading of Recommendations, Assessment, Development and Evaluation (GRADE) was used to evaluate certainty of evidence. Datum and graphs were synthesised using RevMan software, in accordance with Cochrane guidelines.

**Results:**

In total 1,584 participants across the 38 papers involving 49 studies met inclusion criteria. All investigated surgery – 35 papers were case-series and three were cohort studies. No randomised controlled studies were identified. Interventions were sub-grouped as: Endoscopic Repair (*n* = 507), Open Repair (*n* = 950) and Gluteus maximus transfer (*n* = 127). 58% of studies were categorised as low quality, scoring < 65% on the JBI critical appraisal tool. High heterogeneity (clinical and methodological) was observed, and very low certainty of evidence was found according to GRADE assessment. Surgical intervention for people with GTT was associated with improvement of QoL (range, 22.1/100 to 48.8/100) and pain severity (range, -7.0/10 to -1.2/10) at minimum 12-month follow-up.

**Conclusion:**

Very low quality and certainty of evidence found surgical intervention for GTT was associated with improvements in QoL and pain severity. No non-surgical treatments studies for this condition were identified.

**Supplementary Information:**

The online version contains supplementary material available at 10.1186/s12891-026-09519-0.

## Background

Gluteal tendon tears (GTT), affecting the gluteus medius and/or minimus tendons, represent the more severe end of gluteal tendon pathology associated with trochanteric pain, more broadly referred to as Greater Trochanteric Pain Syndrome (GTPS) [1, 2]. Those with symptomatic GTT demonstrate a similar level of disability to that of end-stage hip joint osteoarthritis [1]. Patients commonly experience reduced walking speed and endurance, higher levels of pain, use of pain medication, co-morbidities, and a reduced quality of life (QoL) [1, 3]. This condition exists on a continuum of pathology where gluteal tendinosis is the mild state of the disease [4–7]. This may progress into tendon delamination and fiber disruption such as partial tendon tears, prior to full-tendon tear and/or rupture as the most severe pathological form of the disease [4–8].

The management of GTT comprises both non-surgical and surgical options [9–14], noting that non-surgical management is often described in the literature as first-line management and performed until deemed unsuccessful. However, markers of success or failure remain un-documented [9, 12, 14–17]. These treatments include activity modification, exercise, physiotherapy, corticosteroid injections, platelet-rich-plasma injections, and oral anti-inflammatories [15, 18–21]. Surgical options vary and may depend on tissue/disease status, surgeon training, surgeon preferences and geographical circumstances [15, 22, 23]. Such surgical variations include approach, repair method and the portion of tissue being repaired [24–26].

There have been a number of studies investigating different treatments, particularly surgery [15, 27–29]. The identification of GTT as a possible source of ongoing pain was first noted in the late 1990’s[30]. One of the earliest publications on GTT, was a small retrospective case series of seven patients, who underwent surgical repair [30]. In each case the disrupted tendons re-attached to the greater trochanter using heavy non-absorbable sutures [30]. All patients were reported to be pain-free at a median follow-up of 45 months, however no validated patient reported outcomes measures were used along with no blinding of assessors [30]. A 2015 systematic review examined the outcomes of gluteal tendon repair via open and endoscopic methods [24]. From the eight included studies (*n* = 167), they concluded that endoscopic and open techniques demonstrate comparable positive changes regarding measures of pain, hip abductor strength and disability [24]. However, there was no appraisal on the included studies regarding quality and certainty of findings, nor did they include non-surgical treatments in their search.

Despite GTT being associated with severe pain and disability, research regarding the management of GTT has yet to be synthesised and evaluated for its quality and effectiveness. This raises questions about which treatments for GTT have been investigated or observed and what are the outcomes of these interventions. The purpose of this systematic review was to document treatments, synthesise evidence, and assess the quality of available evidence regarding the treatments and outcomes associated with diagnosed (via clinical examination and confirmed with imaging), symptomatic GTT. The authors hypothesised that both surgical and non-surgical interventions would be effective (as measured by changes in QoL and pain severity) in managing people with GTT.

## Methods

### Design

A systematic review was conducted in accordance to the PRISMA (preferred reporting items for systematic reviews and meta-analyses) guidelines [31]. This systematic review protocol was preregistered on PROSPERO (registration number: CRD42021224593), March 2021. Clinical trial number: not applicable.

### Study inclusion criteria

This review included studies of participants that had a symptomatic partial or full-thickness tear of the gluteus medius and/or minimus tendons, confirmed by imaging (ultrasound or MRI) [32–34]. It included any intervention directed towards improving symptoms and/or repairing tendon tears, in those with symptomatic GTT. Parallel group (comparator) design studies and other designs that reported outcomes before and after an intervention with a mean ≥ 12-month follow-up were included. Primary outcomes of interest were QoL and pain severity. The secondary outcomes were disability, physical function, and adverse events. Papers were required to have a minimum of 10 participants and be written in English. Studies which included > 50% of participants undergoing concomitant intra-articular procedures of the same hip (e.g. labral repair) or participants undergoing total hip arthroplasty of the same hip and/or participants following femur fracture of the same hip were excluded, unless the GTT data was reported separately.

### Search strategy

Databases (MEDLINE, CINAHL, Web of Science, Scopus, Cochrane Library, ClinicalTrials.gov) were searched from their inception until November 2024. The search strategy was as per PROSPERO registration. The search was based on three main concepts: (i) people with GTT, (ii) treatments undertaken and (iii) long-term outcomes. An additional manual search was conducted on the reference lists of previously performed systematic and narrative reviews. Full details of the search strategy are found in Appendix 1.

### Title, abstract and full-text screening

Articles identified through the database search were downloaded into EndNote (Clarivate, Philadelphia, PA, End Note version 20). Files were uploaded into Covidence software (Veritas Health Innovation, Melbourne, Australia (www.covidence.org) with duplicate records removed. Title and abstract screening, followed by full-text screening was performed by two reviewers working independently (TS and AN). Conflicts were resolved independently by a third reviewer (AF).

### Risk of bias assessment

Studies were critically appraised by two authors independently (TS and AN) using the appropriate Joanna Briggs Institute (JBI) critical appraisal tool [35] matched to study design (case-series tool for case-series studies and cohort tool for cohort study designs) [36]. Conflicts were resolved by consensus, with a third independent reviewer (AF) available in the case of disagreements. The scale for the JBI case-series tool ranged from 0 to 10, the cohort study tool ranged from 0 to 11, where the score of 0 represents the highest risk of bias. Data is presented as a percentage with studies scoring ≥ 65% deemed low risk of bias [37].

### Appraisal of the body of evidence

The body of evidence was evaluated using GRADE (Grading of Recommendations, Assessment, Development and Evaluations). The assessment was performed independently by two authors (TS and AN). A third independent reviewer (AF) was available in the case of disagreements. Assessments were applied according to GRADE guidelines [38, 39]. Overall quality of evidence was rated either high, moderate, low or very-low. Quality was first assessed where randomised controlled trials were initially deemed high quality and non-randomised controlled trials deemed low quality/serious risk of bias [40]. Evidence was downgraded for; (i) inconsistency (statistical heterogeneity I^2^ > 40%), (ii) imprecision (< 300 participants for that outcome measure and wide 95% CI where if the true effect was at the lower margin of the CI compared to the upper margin and if this would alter the clinical decision) and (iii) indirectness (generalisation of findings) [41–43]. Large effect size was used to upgrade the level of evidence by one level (standardized mean difference > 1.2). Publication bias (large drop-out (> 20%)) and small case-series (< 20 participants) that affect confidence of findings was used to mark down overall quality [44].

### Data extraction

Two independent authors (TS and AN) extracted data including: study year, population details, participant demographics, eligibility criteria, interventions, outcome measures, follow-up time periods, adverse events, and results. A pre-determined spreadsheet was used to capture data from each study. Data was cross-checked by each author for accuracy. Where insufficient data was provided, the study author(s) were contacted via email on two occasions. Where the author(s) failed to respond, data extraction was confined to the published material. In the event there were two or more publications that used overlapping patient data, preference for data to be included was given to the study with the largest sample population.

For continuous data, mean and standard deviation, median and inter-quartile range or difference in outcomes from baseline was extracted. For dichotomous data, yes/no or positive/negative values were extracted. Where there was missing data, author/s were contacted, where such data was unable to be obtained such as, number of participants at follow-up analysis was performed on the data available.

### Protocol variation

As no randomised controlled trials were identified a meta-analysis was not conducted as this may have unintentionally conveyed an incorrect conclusion and summary of the data.

### Statistical analysis

This study set-out to perform a meta-analysis, however due to the lack of randomised controlled trials a narrative review has been performed.

### Forest plot methods

Where multiple outcome measures that related to a single domain, e.g. disability – Modified Harris Hip Score (mHHS), Harris Hip Score (HHS) and Non Arthritic Hip Score (NAHS) [45], where identified, these measures were pooled, however if these measures were not consistent with one another, preference was given to the outcome measure that was deemed most appropriate for this population and/or captured the largest study population. For example, the reliability and Minimal Clinically Important Change (MCIC) for mHHS has been observed in the GTT population, while the non-arthritic hip score (NAHS) does not have the same appraisal concerning the target population, and thus preference was given to the mHHS in the data synthesis [17].

Data was entered into Review Manager Web ((RevMan Web). Version (5.1). The Cochrane collaboration, available at revman.cochrane.org). Forest plots were created using mean change, with confidence intervals set at 95%. RevMan software, reports ‘mean difference’ which we have interpreted as mean change throughout this review.

Where mean and standard deviations of data were unavailable but a median and interquartile range value was provided, mean and standard deviation was estimated from these values as per the Cochrane handbook [46].

## Results

### Study selection and participant characteristics

**Fig. 1:**
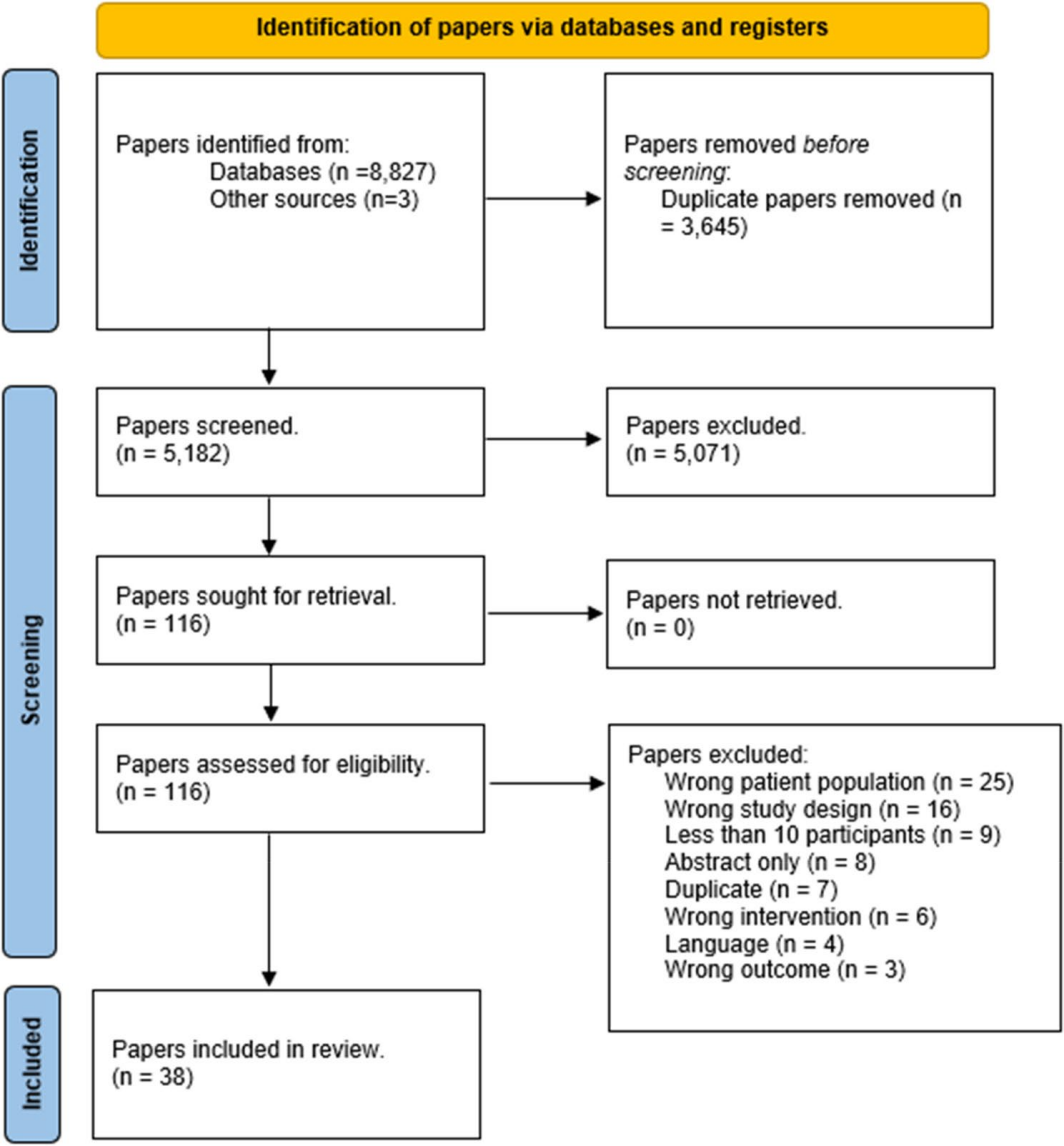
PRISMA flowchart

Following removal of duplicates, 5,182 papers were identified through database searching (Fig. 1). Following screening and full-text reviews, 38 papers were included. Of the 38 papers, 35 (92%) were case series and 3 (8%) were cohort study designs. There were no randomised parallel group design studies found, and no papers investigating non-surgical treatments for GTT. Within this systematic review, we refer to “papers” as the published journal article. Some papers define multiple cohorts, for example Annin, 2021 had identified and separated their studied population into five different cohorts. Each of these cohorts satisfied the current reviews inclusion criteria, of which we refer to these five cohorts as five individual “studies”. As such, this one paper (Annin, 2021) has five studies included in the review. From the included 38 papers, there were 49 studies (*n* = 1,584, 1,411 (89%) female) identified. Of the 49 studies, 40 (82%) had a primary intervention of surgical repair of the gluteal tendon/s (medius and or minimus), while 9 (18%) reported on gluteus maximus/vastus lateralis transfer surgery for the management of GTT.

Across 38 papers and the included 49 studies, various domains were measured using a variety of outcome measurement tools. QoL was measured using the International Hip Outcome Tool (iHOT-12 and/or iHOT-33) in 16 of the 49 studies. Pain severity was measured using Visual Analogue Scale (VAS) in 38 of the 49 studies and Numerical Rating Scale (NRS) in one study. For the domain of disability, the Harris Hip Score (HHS) and the modified version (mHHS) was used in 40 of the 49 studies. For outcomes relating to physical function, 13 studies examined walking gait through visual assessment and eight examined single-leg stance through visual assessment. Of those eight, one study examined single leg stance through 2-D video analysis, where they measured frontal plane mechanics in degrees [47]. Adverse events were reported in 35 studies. The mean duration of follow up of included studies varied from 12 to 78 months.

**Table 1:** Summary of included studies

Study (Author, Year – subgroup if applicable)	*Study design*	*Intervention: EnR (endoscopic repair)*,* OpR (open repair)*,* Transfer (Gmax and/or TFL transfer). Other features*	*Mean age*,* years (SD)*	*Female participants/total participants*	*Patient Reported Outcome Measure/s*	*Measures of Function*	*Adverse events*	*Follow-up period*,* months*	*JBI quality assessment (0–100%)*
Annin, 2021 – FTT DR [15]	Retrospective Case-series	EnR double row repair for full thickness tendon tear	60.2 (12.0)	30/31	mHHS, HOS-SSS, NAHS, iHOT-12, VAS	MMT	1 revision for gluteus medius re-tear	24	80
Annin, 2021 – FTT OpR [15]	Retrospective Case-series	OpR of full thickness tendon tear	66.4 (6.7)	12/13	mHHS, HOS-SSS, NAHS, iHOT-12, VAS	MMT	1 revision for gluteus medius re-tear	24	80
Annin, 2021 – Gmax transfer [15] *Not included in forest plots due to overlap of data with Maldonado*,* 2020* [48]	Retrospective Case-series	Transfer of Gmax tendon	64.1 (15.4)	6/7	mHHS, HOS-SSS, NAHS, iHOT-12, VAS	MMT	No complications	24	80
Annin, 2021 – PTT SS [15]	Retrospective Case-series	EnR of partial tendon tear using suture staples	50.3 (10.2)	18/20	mHHS, HOS-SSS, NAHS, iHOT-12, VAS	MMT	No complications	24	80
Annin, 2021 – PTT TT [15]	Retrospective Case-series	EnR of partial tendon tear using trans tendinous technique	55.6 (9.7)	109/118	mHHS, HOS-SSS, NAHS, iHOT-12	MMT	No complications	24	80
Balazs, 2020 [49]	Retrospective Case-series	OpR, 67% were double row, 33% were single row repairs	51 (range, 17–73)	18/21	mHHS, HOS-ADL, HOS-SSS, iHOT-33	Gait, MMT	2, (1 suffered a fall and subsequent haematoma around surgical site that required surgical intervention, 1 had paraesthesia, all recovered well afterwards).	Mean (range), 48 (24–84)	100
Barrera, 2021 [50]	Retrospective Case-series	OpR suture of gluteus medius full thickness tears	62.4 (18)	13/14	mHHS, VAS	Gait, MMT	No complications	Median (SD), 14.5 (7.0)	80
Bitar, 2023 [51]	Retrospective Case-series	EnR of hip abductor tendon	65 (52;73 IQR)	15/16	mHHS, NAHS (no pre-op measures), VAS	Nil	No complications	Median (range), 24 (3–131)	50
Bucher, 2014 [20]	Prospective Case-series	OpR, with LARS ligament and ‘Y’ TFL decompression and bursal excision	62 (range, 49–74)	19/22	OHS, SF-36, VAS	Nil	2, (1 had complication with catheter requiring further surgical intervention, 1 had irritation due to implanted screw holding LARS ligament).	12	70
Christofilopoulos, 2020 – native hip [26]	Retrospective Cohort	Transfer of Gmax flap	65.9 (5.6)	8/10	HHS, VAS	Gait, MMT	12 patients across the entire cohort of 38 had seroma of their wound. Symptoms subsided within a few days.	Mean (SD), 19.2 (9.1)	36
Christofilopoulos, 2020 – post primary THA [26]	Retrospective Cohort	Transfer of Gmax flap following primary THA	66.3 (4.7)	4/6	HHS, VAS	Gait, MMT	Refer to above Christofilopoulos 2020.	Refer to above Christofilopoulos 2020.	36
Christofilopoulos, 2020 – post revision THA [26]	Retrospective Cohort	Transfer of Gmax flap following revision THA	73.2 (3.5)	17/22	HHS, VAS	Gait, MMT	Refer to above Christofilopoulos 2020.	Refer to above Christofilopoulos 2020.	36
Coulomb, 2016 [21]	Retrospective Case-series	EnR via performing micro-perforation in the enthesis	53.5 (13.8)	16/17	HHS, VAS	SLS, gait	3 patients had incision pain, 1 experienced snapping of hip 26 months post-surgery with poor outcome.	Mean (SD), 37.6 (10.4)	30
Davies, 2009 [52]	Prospective Case-series	OpR using DR sutures re-attaching tendons onto trochanteric footprint	63 (range, 47–82)	15/16	OHS, SF-36, MD, VAS (no variability reported)	Nil	4 had re-tears within follow-up period, 3 of these were revised, with another subsequent re-tear. 1 experienced a deep wound infection requiring debridement. *All complications were removed from follow-ups	12	20
Davies, 2013 [53]	Retrospective Case-series	OpR with treatments ranging from sutures, double row repair and autograft as they relate to tissue quality	67.7 (range, 45–85)	20/22	mHHS, LEFS	Nil	No complications	Mean (range), 70.8 (61–100)	60
Della Rocca, 2022 [54]	Retrospective Case-series	EnR of gluteus medius full thickness tears	58.6 (4.9)	18/22	mHHS, HOS-ADL, HOS-SSS, LEFS, VAS	Nil	1 participant had re-operation for a THA	Mean (range), 42 (24–72)	70
Ebert, 2020 [28]	Prospective Case-series	OpR, bursectomy, VY lengthening and re-attachment of tendon with LARS ligament	64.3 (range, 43–84)	132/144	HHS, OHS, SF-12, VAS	SLS, 6MWT, strength (kg/f)	8 surgical failures where patients re-presented with similar symptoms prior to surgery. 3 of these people underwent revision. 1 developed Deep Vein Thrombosis that perpetuated into a Pulmonary Embolism 3 weeks post-op. 4 with superficial wound infections and 1 with wound hematoma all of which resolved	24	80
Ebert, 2018 [55] *Not included in forest plots due to overlap of data with overlap of data with Ebert*,* 2020*	Prospective Case-series	OpR, bursectomy, VY lengthening and re-attachment of tendon with LARS ligament	63.2 (range, 43–82)	101/110	HHS, OHS, SF-12, VAS	SLS, 6MWT, strength (kg/f)	3 surgical failures with re-appearance of pre-operative symptoms and re-tear of tendon. 1 patient developed a haematoma post-operatively, 2 had superficial wound infection, 1 developed Deep Vein Thrombosis that perpetuated into a Pulmonary Embolism 3 weeks post-op	12	80
Ebert, 2019 [56] *Not included in forest plots due to overlap of data with Ebert*,* 2020*	Retrospective Case-series	OpR, bursectomy, VY lengthening and re-attachment of tendon with LARS ligament	64.6 (range, 43–84)	84/84	HHS, OHS, SF-12, VAS	SLS, 6MWT, strength (kg/f)	2 superficial wound complications, 1 post-operative haematoma, 3 failures (defined as presenting with post-operative pain within 2 years), re-tear was confirmed in all 3	24	90
Fink, 2018 [57]	Retrospective Case-series	OpR using sutures with non-resorbable collagen patch	76.8 (4.3)	28/30	HHS, VAS	MMT	No complications	12	40
Fox, 2020 [58]	Retrospective Case-series	OpR, mix of partial and full-tendon tears using suture repair	69 (range, 34–91)	152/165	OHS	Nil	Deep vein thrombosis occurred in 4% of participants.	Mean (range), 78 (60–120)	40
Groot, 2011 [59]	Retrospective Case-series	OpR, Tendons reattached with transosseous steel wires and mattress suture technique. All following primary THA	63 (range, 46–76)	13/15	Nil	Nil	No complications	Mean (range), 34 (6–77)	20
Hartigan, 2018 [60] *Not included in forest plots due to overlap of data with Annin*,* 2021.*	Retrospective Case-series	EnR, trans tendinous repair of partial to complete tendon tears	53.5 (range, 38.4–70.7)	24/25	mHHS, HOS-ADL, HOS-SSS, NAHS, VAS	Gait	No complications	Mean (range), 38 (26.6–68)	80
Huxtable, 2017 [47] *Functional measure of SLS was utilised in forest plots. All other data not included in forest plots due to overlap with Ebert*,* 2020*	Prospective Case-series	OpR, bursectomy, VY lengthening and re-attachment of tendon with LARS ligament	62.1 (7.9)	19/21	HHS, SF-12, OHS, VAS	SLS (two-dimensional measures), 6MWT, strength (kg/f)	Not reported	12	70
Kirby, 2020 – FTT [61]	Retrospective Case-series	EnR with non-absorbable sutures and bio composite anchors	46.0 (11.4)	7/8	mHHS, NAHS	Gait	No complications	Mean (SD), 30 (12.8)	80
Kirby, 2020 – PTT [61]	Retrospective Case-series	EnR with non-absorbable sutures and bio composite anchors	54.8 (11.3)	8/12	mHHS, NAHS	Gait	No complications	Mean (SD), 28 (10.7)	80
Kocaoglu, 2021 – DR [22]	Retrospective Cohort	EnR using double row repair	53.9 (8.9)	13/16	mHHS, HOS-ADL, HOS-SSS, VAS	Nil	Not reported	Mean (range), 30 (24–42)	64
Kocaoglu, 2021 – SR [22]	Retrospective Cohort	EnR using single row repair	52 (6.9)	12/14	mHHS, HOS-ADL, HOS-SSS, VAS	Nil	Not reported	Mean (range), 30 (24–42)	64
Kocaoglu, 2021 – SR + MF [22]	Retrospective Cohort	EnR using single row repair and microfracture	54 (8.3)	16/20	mHHS, HOS-ADL, HOS-SSS, VAS	Nil	Not reported	Mean (range), 30 (24–42)	64
Kohl, 2012 [62]	Retrospective Case-series	Transfer of vastus lateralis post THA	65 (no SD reported)	5/11	MD	MMT	3 complications: 1 had fibular nerve palsy, 1 had Complex Regional Pain Syndrome post-op, 1 had revision for insufficiency of Vastus Lateralis	24	40
Lemme, 2023 [63]	Prospective Case-series	Gmax transfer procedure	69 (9.2)	17/21	mHHS, HOS-ADL, HOS-SSS, VAS	MMT	1 developed deep infection of a seroma, no other events identified	12	90
Makridis, 2014 [64]	Retrospective Case-series	OpR using anchors and sutures. Mix of partial and complete tendon tears and varying degrees of fatty infiltration.	Median = 68 years (range, 25–87)	62/67	HHS, VAS	Nil	Not reported	Mean (range), 55.2 (12–96)	70
Maldonado, 2020 [27] *Not included in forest plots due to overlap of data with Annin*,*2021*	Retrospective Case-series	OpR, of full-thickness glute medius tears.	65.2 (12.7)	31/36	mHHS, HOS-SSS, HOS-ADL, iHOT-12, NAHS, VAS	MMT	1 patient converted to THA 48.2 months after tendon repair procedure	Mean (SD), 40.8 (26.19)	60
Maldonado, 2020 #2 [48]	Retrospective Case-series	Transfer of Gmax and Tensor Fascia Latae	68.5 (11.1)	13/18	mHHS, HOS-SSS, HOS-ADL, iHOT-12, NAHS	MMT	Not reported	Mean (range), 39.75 (12.04–93.88)	60
Miozzari, 2010 [65]	Retrospective Case-series	OpR through lateral approach reattaching avulsed abductor insertion/s following THA	62.2 (11.2)	8/12	HHS, VAS	SLS, gait, MMT	2 had recurrence of tear on MRI	Mean (range), 27.8 (12.5–54.7)	40
Nazal, 2020 [19]	Prospective Case-series	EnR of partial and complete tendon tears	66.9 (9)	12/15	mHHS, HOS-SSS, HOS-ADL, NAHS, iHOT-33, VAS	MMT	No complications	Mean (SD), 31.2 (10.92)	50
Paul, 2024 – Lumbar pathology [66]	Retrospective Case-series	EnR of gluteus medius, in presence of people with lumbar pathology	65.2 (7.7)	17/19	iHOT-12, HOS-ADL (no pre-op data), VAS	Nil	Not reported	Mean (range), 27.6 (6–60)	60
Paul, 2024 – Without lumbar pathology [66]	Retrospective Case-series	EnR of gluteus medius, in people without lumbar pathology	61.0 (9.1)	3/4	iHOT-12, HOS-ADL (no pre-op data), VAS	Nil	Not reported	Mean (range), 27.6 (6–60)	60
Portet, 2024 [67]	Retrospective Case-series	Gmax transfer by either open or endoscopic techniques	74 (66; 76 IQR)	11/14	mHHS, iHOT-12, NAHS, VAS	SLS, MMT	Not reported	Median (range), 41 (12–59)	70
Rao, 2012 [68]	Prospective Case-series	OpR using graft jacket/augment	68 (range, 62–84)	8/12	HHS (no SD provided)	Gait, SLS, MMT	1 post-operative hematoma that required drainage	Mean (range), 22 (15–34)	50
Ratnayake, 2020 [69]	Retrospective Case-series	OpR of avulsions and tears using a mix of anchors and tunnels for repair	63 (10)	22/27	HHS, VAS	Nil	No reported	12	40
Ruckenstuhl, 2020 [70]	Retrospective Case-series	Transfer of Gmax post THA and severe hip abductor insufficiency	64 (53–79)	15/18	HHS, SF-36, HRQoL, NRS	MMT	Not reported	Mean (range), 33.2 (8–59)	50
Saltzman, 2018 – without PRFM [12]	Retrospective Cohort	EnR on mix of fatty infiltrations levels without PRFM	63.1 (12.0)	25/29	HHS, mHHS, HOS-ADL, HOS-SSS, iHOT-12, SF-12, VAS	Nil	Not reported	12	64
Saltzman, 2018 – with PRFM [12]	Retrospective Cohort	EnR on mix of fatty infiltrations levels with PRFM	60.3 (8.8)	16/18	HHS, mHHS, HOS-ADL, HOS-SSS, iHOT-12, SF-12, VAS	Nil	Not reported	12	64
Saltzman, 2019 [71] *Some data not included in forest plots due to overlap with Saltzman*,* 2018*	Retrospective Case-series	EnR on mix of fatty infiltration levels	59.3 (10.1)	44/47	mHHS (used 2018 data), HOS-ADL, HOS-SSS, iHOT-12, VAS (used 2018 data)	Nil	Not reported	Mean (range), 37.4 (18–64)	60
Thaunat, 2021 [72]	Prospective Case-series	EnR of isolated partial and full-thickness tears using sutures and anchors	62.7 (9)	43/46	mHHS, NAHS, VAS	Nil	4 had failure of repair, 1 of those underwent revision with unsatisfactory outcome. The other three were managed satisfactorily conservatively.	24	80
Uppstrom, 2021 [17]	Retrospective Case-series	OpR of partial and complete tears using sutures and anchors	63.7 (10.7)	37/47	mHHS, iHOT-33	Nil	4 patients endured post-operative complications.	Mean (SD), 37.8 (27.9)	80
Voos, 2009 [16]	Retrospective Case-series	EnR with sutures, half were partial and half were full-thickness tears	50.4 (range, 33–66)	8/10	HOS, VAS	MMT	No Complications	Mean (range), 25 (19–38)	50
Walsh, 2011 [23] *Not included in forest plots due to overlap of data with Fox*,* 2020*	Retrospective Case-series	OpR with sutures, mix of partial and full-thickness tears	62 (range, 36–88)	67/72	Relief of pain and MD	Nil	19% complication rate: 6 had deep vein thrombosis, 1 had pulmonary embolism, 1 had pressure sore, 3 had wound haematoma, 4 had tendon re-tear within 6 weeks of surgery, 1 had greater trochanter fracture and 1 had wound infection	12	30

Scores ≥ 65% for JBI quality assessment are deemed low risk of bias

*6MWT* Six minute walk test, *ADL* Activities of Daily Living, *DR* Double row, *EnR* Endoscopic repair, *FTT* Full tendon tear, *Gmax* Gluteus Maximus, *HHS* Harris Hip Score, *HRQoL* Hip related quality of life, *HOS* Hip outcome score, *iHOT* International hip outcome tool, *LARS* Ligament augmentation reconstruction system, *LEFS* Lower extremity functional scale, *MD* Merle d’Aubigne hip score, *MF* microfracture, *mHHS* Modified Harris Hip Score, *MMT* Manual muscle testing, *NAHS* Non-arthritic hip score, *NRS* Numerical Rating scale for pain severity, *OHS* Oxford hip score, *OpR* Open repair, *PTT* Partial tendon tear, *PRFM* Platelet rich fibrin matrix, *SD* Standard deviation, *SF* 12/36–12/36-item short form survey, *SLS* Single leg stance, *SS* Suture staple, *SSS* Sport specific scale, *THA* Total hip arthroplasty, *TT* Transtendinous, *VAS* Visual Analogue scale for pain severity

### Risk of bias

Studies were assessed using the JBI critical appraisal tool, 16 of the 38 papers scored ≥ 65% (Table 1). Common reasons for marking down the risk of bias score for the case-series studies included not using valid methods to identify the condition, not having reported consecutive inclusion of participants, incomplete inclusion of participants and absent reporting of clinical information of participants. Reasons for marking down the quality of included cohort studies were; failure to identify and account for confounding factors, and inappropriate analysis to deal with incomplete patient follow-up.

### Summary of GRADE findings

The certainty of evidence was deemed very low for all measures (Table 2): QoL (iHOT-12/33), pain severity (VAS), disability (mHHS/HHS) and hip abductor strength (MMT) [38, 39]. Certainty of evidence was consistently downgraded due to study design, risk of bias and inconsistency.

**Table 2:** Summary of findings table using GRADE to mark the certainty of evidence (Guyatt et al., 2008).

Outcome	Number of studies (participants)	Study design	Risk of bias	Inconsistency	Indirectness	Imprecision	Other considerations	Overall effect (SMD (95%CI))	Certainty of evidence (GRADE)
Measure of Quality of Life (iHOT-12/33) (mean ≥ 12 months)	9 (321)	Non-RCT	Serious	Serious	Not serious	Not serious	Strong association and undetected publication bias	NA*	VERY LOW
Measure of pain severity (VAS) (mean ≥ 12 months)	31 (847)	Non-RCT	Serious	Serious	Serious	Not serious	Strong association and undetected publication bias	NA*	VERY LOW
Measures of disability (mHHS/HHS) (mean ≥ 12 months)	31 (883)	Non-RCT	Serious	Serious	Not serious	Not serious	Strong association and undetected publication bias	NA*	VERY LOW
Measure of hip abductor strength (MMT) (mean ≥ 12 months)	18 (418)	Non-RCT	Serious	Serious	Serious	Serious	Undetected publication bias	NA*	VERY LOW

*GRADE* Grading of Recommendations, Assessment, Development and Evaluation, *HHS* Harris Hip Score, *iHOT *International Hip Outcome Tool, 12/33, *mHHS* modified Harris Hip Score, *MMT *Manual Muscle Testing, *RCT *Randomised Controlled Trial, *SMD S*tandardised Mean Difference, *VAS *Visual Analogue Scale

NA* - Overall effect size not reported as no pooled results

## Results

### Quality of life

**Fig. 2:**
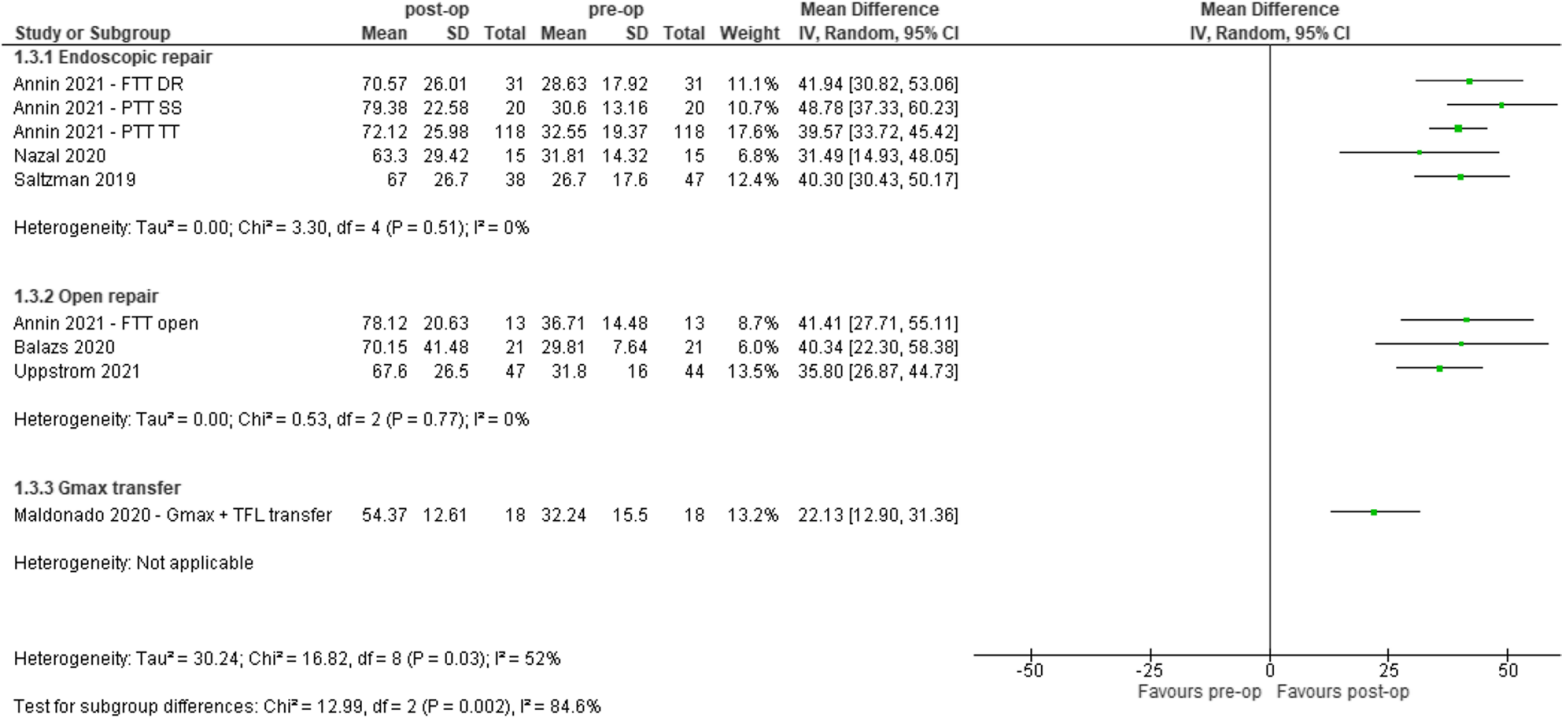
Pre-and post-op outcomes for quality of life (International Hip Outcome Tool – 33 or 12, 0–100), CI – confidence interval, DR – double row, FTT – Full-thickness tear, Gmax – Gluteus maximus, IV - inverse variance, PTT – partial thickness tear, SS – suture staple, TFL – Tensor Fascia Latae, TT – transtendinous

Nine out of the 49 (18%) studies were included in the analysis for QoL. People who underwent surgical reconstruction of GTT had improved QoL at mean ≥ 12 months, as measured by iHOT-12/33 (0–100 scale) post-operatively compared to pre-operatively (Range: 22.13 to 48.78). All studies showed positive effects, Fig. 2.

### Pain severity

**Fig. 3:**
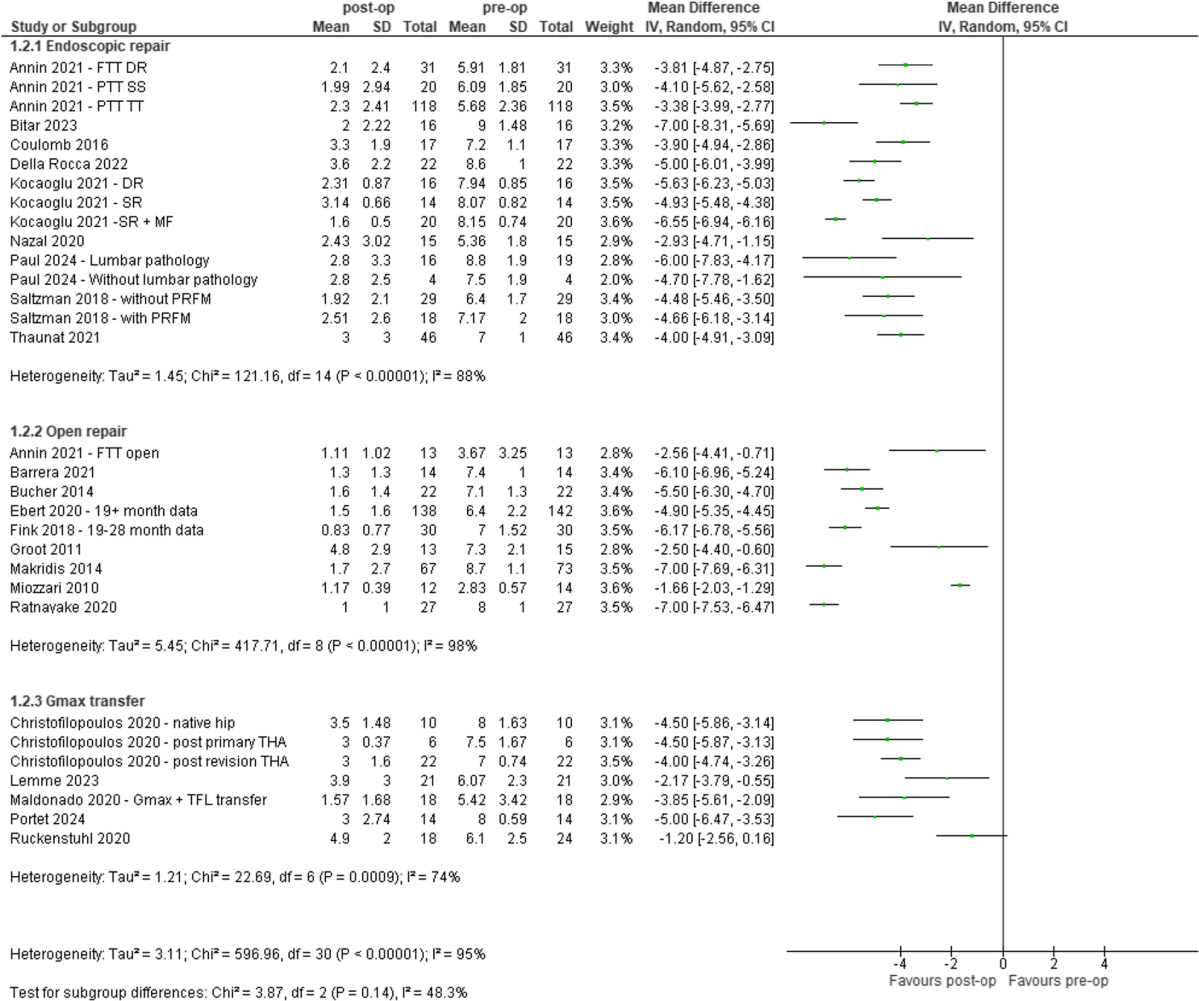
Pre-and post-op outcomes for pain severity (Visual Analogue Scale, 0–10). CI – confidence interval, DR – double row, FTT – Full-thickness tear, Gmax – Gluteus maximus, IV - inverse variance, MF – Micro-fracture, PRFM – Platelet Rich Fibrin Matrix, PTT – partial thickness tear, SR – single row, SS – suture staple, TFL – Tensor Fascia Latae, THA – total hip arthroplasty, TT – transtendinous

Thirty-one out of the 49 (63%) studies reported pain severity and were included in the analysis. People who underwent surgical reconstruction of GTT had lower pain severity at mean ≥ 12 months, as measured by VAS and NRS (0–10 scale), post-operatively compared to pre-operatively (Range: −7.0 to −1.2). One study showed no effect, Fig. 3.

### Disability

**Fig. 4:**
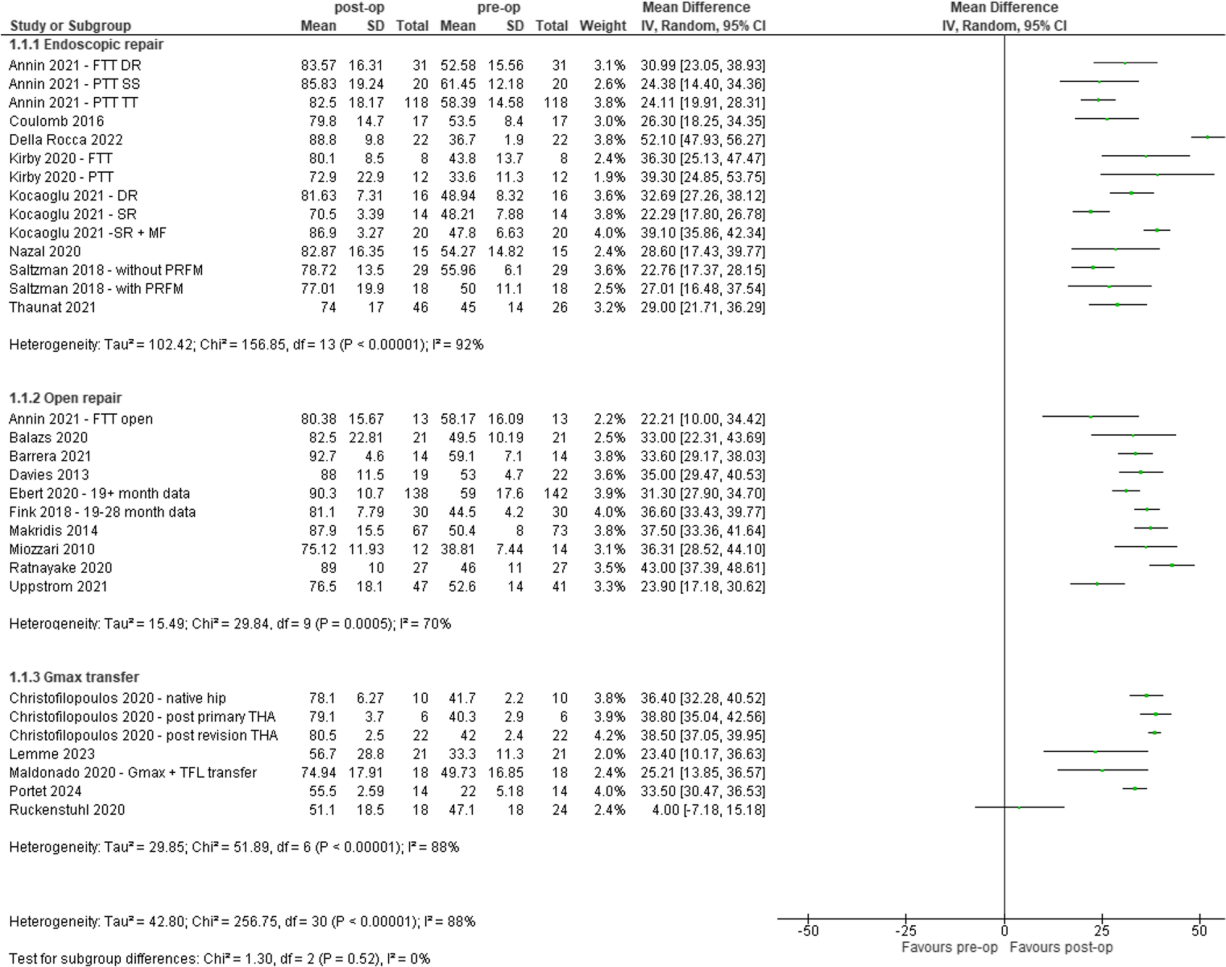
Pre- and post-op outcomes for disability (Modified Harris Hip Score or Harris Hip Score, 0–100). CI – confidence interval, DR – double row, FTT – Full-thickness tear, Gmax – Gluteus maximus, IV - inverse variance, MF – Micro-fracture, PRFM – Platelet Rich Fibrin Matrix, PTT – partial thickness tear, SR – single row, SS – suture staple, TFL – Tensor Fascia Latae, THA – total hip arthroplasty, TT – transtendinous

Thirty out of the 49 (61%) studies were included in the analysis for disability. People who underwent surgical reconstruction of GTT had reduced levels of disability, as measured by mHHS and HHS (0–100 scale) at mean ≥ 12 months, post-operatively compared to pre-operatively (Range: 4.0 to 52.1). One study showed no effect, Fig. 4.

### Hip abductor strength

**Fig. 5:**
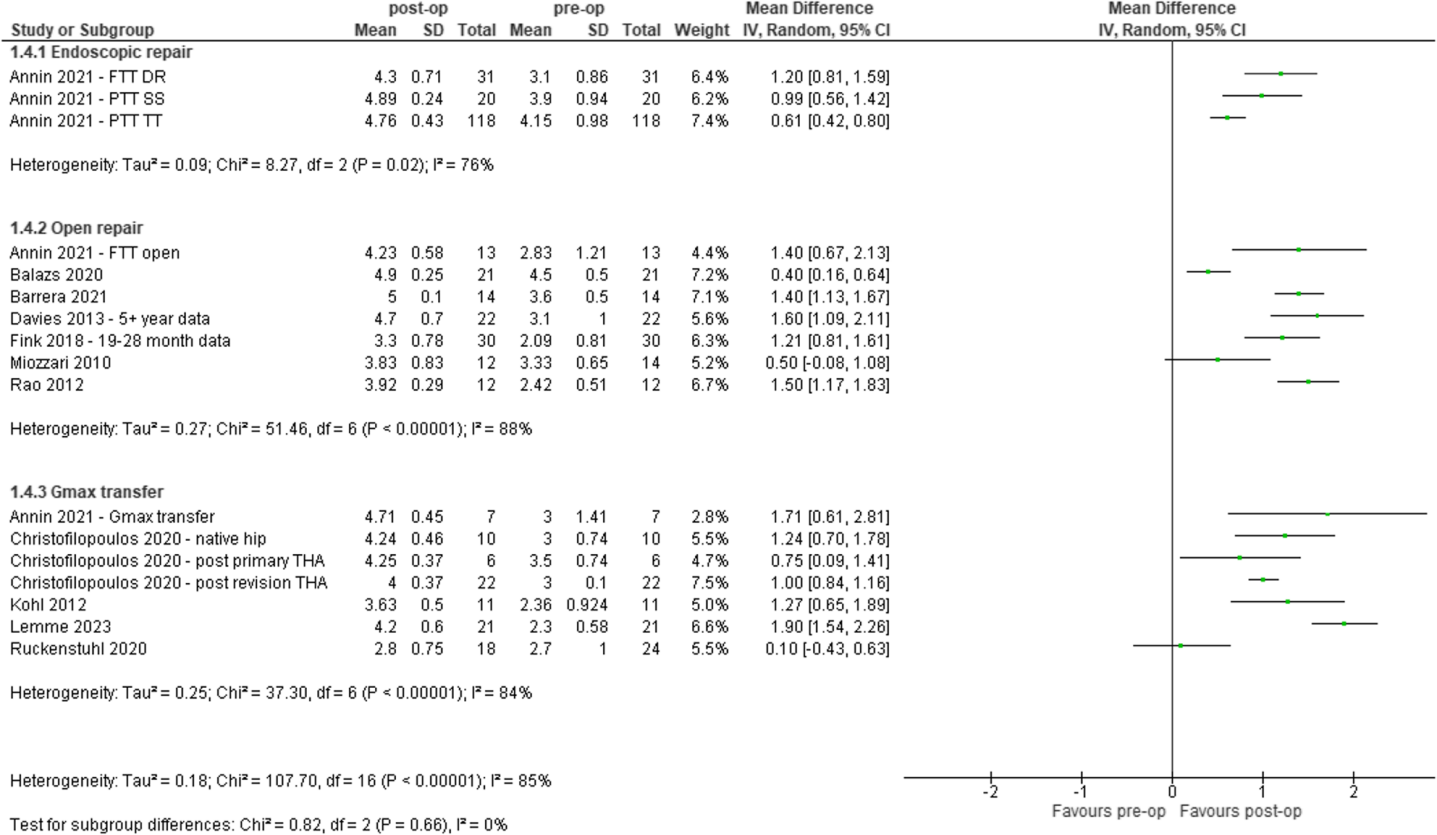
Pre- and post-op strength assessments (Manual Muscle Testing, 0–5). CI – confidence interval, DR – double row, FTT – Full-thickness tear, Gmax – Gluteus maximus, IV - inverse variance, PTT – partial thickness tear, SS – suture staple, THA – total hip arthroplasty, TT – transtendinous

Seventeen out of the 49 (35%) studies in the analysis recorded measures for hip abductor strength using manual muscle testing. Results show people who underwent surgical reconstruction of GTT had improved hip abductor strength, as measured by manual muscle testing (0–5 scale) at mean ≥ 12 months, post-operatively compared to pre-operatively (Range: 0.10 to 1.90). Two studies showed no effect, Fig. 5.

### Adverse events

There was inconsistent reporting of adverse events. Of the 49 studies, 14 (29%) did not report adverse events. Of the 35 studies reporting adverse events, 13 reported that no significant adverse events occurred. The remaining 22 studies reported complications including: re-tear of gluteal tendon/s, greater trochanter fracture, wound hematoma, wound infection, deep vein thrombosis, pulmonary embolism, revision surgery, pressure sore, peroneal nerve palsy, complex regional pain syndrome, conversion to total hip arthroplasty, seroma, catheter complication requiring surgery, incision pain, paresthesia and a subsequent post-operative fall which required further surgery (Table 1).

Re-tear rates ranged from 8.7%[72] to 16.7%[65]. In a particular study, four out of the 46 participants experienced a re-tear, however only one had revision surgery whilst the other three were satisfactorily managed through non-surgical management [72]. For context, two systematic reviews examined retear rates in shoulder rotator cuff repair and found that incidence of re-tears ranged from 27.1% to 35%[73, 74].

The rate of revision surgery varied from 2%[28] to 25%[52]. One study reported an incidence of 5.5% of participants developing pre-surgical symptoms, however only 2% of these participants underwent revision procedures [28].

Three studies reported venous thrombotic events that ranged in incidence from 0.7%[28] to 8.3%[58]. One study had a 4% rate of deep vein thrombosis complications in their participant group of 165 undergoing open repair [58]. Another study reported 6 deep vein thrombotic events and 1 pulmonary embolism event in their participant group of 72 participants, representing a rate of 8.3% (deep vein thrombosis) and 1.3% (pulmonary embolism) respectively [23]. For added context, a large retrospective study of 29,264 patients examined venous thrombotic events following hip or knee arthroplasty surgery, they observed the cumulative incidence of venous thrombotic events at 30 days as 1.19%[75].

Gluteus maximus and vastus lateralis transfer procedures were performed for irreparable gluteus minimus and or medius tears. These procedures had a varied incidence of adverse events ranging from 0%[15] to as high as 32%[26] which all related to development of wound seromas. One study observed a 27% incidence of adverse events which included complex regional pain syndrome, fibular nerve palsy and vastus lateralis insufficiency post-op, these were unique to this study alone [62].

### Trendelenburg sign during gait and single leg stance

Thirteen studies reported on gait and eight reported on single-leg stance, often by referring to it as Trendelenburg gait or Trendelenburg stance [76]. A pre-post visual assessment by an unblinded assessor using a categorical grading systems was common, e.g. assessing the severity of a Trendelenburg sign as not present, mild, moderate, major, or unable to stand on a single leg [21], or dichotomously as “present” or “not present” [18].

Of the thirteen studies that reported on gait, all studies stated improvements post-operatively of varying magnitude. Presence of Trendelenburg gait pre-operatively ranged from 10%[61] to 100%[65, 68]. Resolution of a pre-operative Trendelenburg gait ranged from 50% (half had persistent Trendelenburg gait) [21] to 100% (all participants had a resolved Trendelenburg gait) [18].

Of the eight studies that reported on single leg stance, one study, Huxtable et al. (2017), quantified the presence of Trendelenburg sign during a single leg stance test. They used 2D motion capture technology to assess changes in pelvis on femur angle, pelvic drop, trunk lean and lateral pelvic shift [47]. Huxtable et al. (2017) noted 24% of participants demonstrated a Trendelenburg sign, observed through visual assessment. At post-operative follow-up, 5% of participants had a persistent Trendelenburg sign post-surgery [47]. In contrast, the 2D measures performed on the same population demonstrated no significant changes from pre-op to post-op[47]. Another study examined single leg stance over 30 s, recording pain at 10 s intervals. There was significant improvement in pain at 24 month follow-up[28]. Among other studies that used visual assessment only, Miozzari (2010) observed 100% of participants (*n* = 12) showed a Trendelenburg sign. Post-surgery, 10 participants had complete resolution, with 2 participants continuing to demonstrate a Trendelenburg sign [65]. Similar was observed with Rao et al. (2012), who had all 12 participants present with a positive Trendelenburg sign. Post-surgery, 1 of the 12 patients still demonstrated a persistent Trendelenburg sign on testing [68].

### Hip abductor strength

Twenty-three studies reported on hip abductor strength. Of these, 19 used manual muscle testing which is a categorical, subjective measurement by the examiner ranging from 0 – no muscle contraction to 5 – full muscular power against gravity and resistance [77]. Four studies by the same author and research group used fixed dynamometry to quantify maximal isometric hip abductor strength [28, 47, 55, 56]. Limb symmetry index was used to standardise hip abductor strength to the unaffected side. Pre-surgery, limb symmetry index (SD) was 90.1% (42.5), favoring the non-operative limb. At 12 months, limb symmetry index improved to 105.8% (27.0) and 24 months was 102.6% (15.0), in favor of the operative limb [28]. Notably, data from 8 participants with reported surgical failures were omitted from the results, as was the data from an additional 14 participants with bilateral greater trochanteric pain syndrome.

### Walking endurance

One study examined walking endurance objectively using the six-minute walk test [56]. Pre-surgery, the mean (SD) distance covered by participants was 421.8 m (91.9) with a self-reported pain severity of 4.7/10 (2.7) (VAS at end of six-minute walk test). Twenty-four months post-operatively, participants covered a mean (SD) of 509.7 m (105.0) with a pain severity of 1.0/10 (1.5). Both distance walked and pain severity improved significantly [28].

## Discussion

This review identified 38 eligible papers. No randomised parallel group designs were identified. Papers examining non-surgical interventions were not identified, despite literature in this area frequently suggesting it’s use [9, 15, 21, 22]. This review found very low-quality evidence for gluteal tendon surgery in the management of GTT in improving QoL, pain severity, disability and hip abductor strength at mean ≥ 12 months. Notwithstanding this, improvements observed by majority of studies exceeded the previously noted MCIC values for this population regarding QoL (iHOT-33) and disability (mHHS) [17]. The review reports a re-tear rate of between 8.7%[72] to 16.7%[65].

### Study quality

Overall, the quality of the evidence included in this review was very low due to the low methodological quality of the studies that have investigated the short to long-term outcomes for this population. No randomised controlled studies, or even any comparative studies were identified. Thus no meta-analysis was able to be performed. The majority of included studies lacked the rigour of a well-conducted observational design. Common issues were selection, sampling, assessor and attrition bias. Other problems included non-consecutive participant inclusion, incomplete data recording and inappropriate statistical analysis. Thus, it is inappropriate to make strong clinical recommendations from the studies and their data alone.

### Heterogeneity

There was a high level of heterogeneity identified in the data synthesis (I² >40%). This could be explained by both the occurrence of clinical and methodological heterogeneity. The observed clinical heterogeneity included: different and variable population groups that varied on disease stage, age of participants and varying types of surgical procedures (e.g., LARS repair, single-row repair, double-row repair, gluteus maximus transfer). Types of methodological heterogeneity observed included non-blinding of assessors, failure to report dropouts or lack of intention to treat analysis, variable reporting on adverse events and non-consecutive inclusion of participants being common factors. The heterogeneity limits accuracy, consistency and the overall clinical implications that can be drawn from this review. With these strengths and limitations in mind, this review offers significant insight into the sources of variability and sub-group analysis (Endoscopic Repair, Open Repair, and Gluteus Maximus/Vastus Lateralis transfer) that can further understanding within this field.

### Surgical interventions

A wide range of surgical interventions and variations were identified and included in this review. These were categorised into three groups: endoscopic repair, open repair and gluteus maximus/vastus lateralis transfer. It is understood that selection of surgical technique may differ for a variety of reasons including disease state, location of pathology, surgeon preference, surgeon training and patient characteristics. Whilst these differences between groups exist, there were improvements observed across measures of QoL, pain, disability and hip abductor strength. All studies except one surpassed the mHHS MCIC (9.9 points) and iHOT-33 MCIC (14.3 points) for open gluteus medius or minimus tendon repair [17, 70].

### Non-surgical interventions

This review hypothesised that non-surgical interventions would be effective in managing people with GTT. However, no literature capturing (a) natural history, (b) non-surgical management or (c) comparison between surgical, non-surgical and/or sham treatments was identified. This is despite numerous references to participants trialing non-surgical management pathways (physical therapy, NSAIDs, corticosteroid injections), with a period of 1–6 months being common [12, 15, 16, 19, 22, 54, 78]. No data was available from the included studies that examined how many participants underwent non-surgical management with satisfactory outcome and subsequently withdrew or opted out of surgical management. One reference to participants receiving a satisfactory outcome via non-surgical management was when four participants were identified as having a re-tear of their tendons and reappearance of symptoms post-operatively [72]. Three of the four participants were reported to have been managed satisfactorily through non-surgical management, but no description of what this management entailed was provided [79]. Another study, examined prediction rules of those more likely to undergo surgery or not in the presence of symptomatic full-thickness GTT [9]. All participants received 3 months of physical therapy, gait re-training and an option of corticosteroid injection [9]. From this study, 12 (33%) participants showed improvements and were discharged from care [9]. However, no detail of the non-surgical management was provided, no long-term (12 months or more) follow-up was undertaken and no patient reported outcome measures were reported [9]. While GTT literature often cites exhaustion of non-surgical management (exercise therapy, corticosteroid injections etc.) before undergoing surgical intervention, gluteal tendinopathy literature has investigated non-surgical management through randomised controlled trials [14, 80]. From gluteal tendinopathy literature, favorable outcomes have been shown with exercise and education [14, 80], shock wave therapy [81], platelet-rich plasma injection [11, 82], pharmaceuticals [83], and corticosteroid injections [81, 84, 85]. Given that favorable outcomes have been observed in patients with gluteal tendinopathy, such treatments could be assessed for safety and efficacy in GTT populations. It is a pertinent reminder that non-surgical management trials within the GTT literature is a key evidence gap reducing the ability to inform clinical decision making for both practitioner and patient.

### Previous reviews in the area

Chandrasekaran et al.’s (2015) systematic review (k = 7, *n* = 167) examined outcomes related to open and endoscopic repairs of GTT. They found no significant differences due to surgical technique on post-operative outcomes of hip abductor strength, pain, and disability, as both groups improved significantly from pre-operative status [24]. The authors observed that open procedures had a higher complication rate than endoscopic procedures, reporting the open repair group had a re-tear rate of 10 in 128 patients and the endoscopic group had 0 re-tears in 35 patients [24]. This finding is likely confounded by numerous factors; the open repair group mean age was higher than the endoscopic repair group, fewer numbers were observed in the endoscopic repair group and a wide variation in follow-up time periods existed between groups. Additional confounding variables included, treatment of concomitant intra-articular pathology, study design limitations and the inability to ascertain similar levels of disease and disability between the groups due to insufficient patient characteristics and outcome measures reported by the included studies [24]. Other known prognostic factors that have been associated with poorer outcomes such as, longer duration of symptoms, larger number of corticosteroid infiltrations and smoking status, were not captured or reported [86]. This current review (k = 49, *n* = 1584) supports the previous reviews’ findings that there is no apparent difference between surgical technique groups across pain, disability, abductor strength and QoL for either open or endoscopic approaches due to the considerable overlaps of their respective 95% confidence intervals [24]. Regarding adverse events, this current review finds it inappropriate to suggest that one surgical approach has fewer adverse events than the other. This is due largely to varying patient demographics, follow-up time periods and inconsistency of reporting.

To enhance clinical guidelines and recommendations for this population high quality research is required. Firstly, there is a need to investigate the natural history of symptomatic GTT, the prevalence of GTT and the incidence/rate by which people diagnosed undergo surgical procedures. Importantly there is a need to determine the proportion of people diagnosed who do not undergo surgery, along with the long-term outcomes. Secondly, well performed randomised controlled trials of various management strategies are needed to determine the efficacy of treatments. The authorship team believes that answering these questions will help build confidence in decision making for both patient and practitioner as well as improving outcomes for those affected by this debilitating and poorly understood condition.

### Strengths and limitations

This is the first systematic review of the management of GTT and included 1,584 participants in the analysis. There were several limitations to this systematic review. Most significantly, there were no randomised controlled trials which detracted from the strength of the findings and ability to perform a meta-analysis and thus determine treatment effectiveness. This review was set up to include < 50% of participants undergoing concomitant intra-articular procedures to be in accordance with the Cochrane Handbook (Sect. 3.2.2)[46]. It is important to note that from the included papers, no papers included concomitant intra-articular procedures, therefore reducing confounding variables and further sources of heterogeneity. Additionally, our eligibility criteria required studies to self-identify as investigating GTT Studies on gluteal tendinopathy were excluded, even though some may have incidentally included participants with GTT. A recent study found that 42% of patients in a trial investigating exercise on gluteal tendinopathy had GTT [87]. It is important to acknowledge the potential for sampling bias within this study. Patients who responded well to non-surgical management and did not progress to surgical intervention are unlikely to be captured, leaving a cohort that may represent those with more persistent symptoms – this could have influenced outcomes and limit generalisability. The data synthesis was restricted to the outcome measures that were most frequently used and had prior MCIC values associated with the studied population. The outcome measures commonly used had largely been developed for hip arthroplasty and hip osteoarthritis, there was no use of a valid or sensitive outcome measurement tool developed and validated for the gluteal tendon pain population [88–91].

## Conclusion

The quality and certainty of evidence for the surgical management of GTT is very low. However, surgical intervention for GTT was associated with improvements in QoL and pain severity. No evidence was identified relating to non-surgical treatments for this condition. Furthermore, no randomised controlled trials were identified. Together this demonstrates a significant evidence gap in the management of this population.

## Supplementary Information

Supplementary Material 1.

## Data Availability

All data supporting the findings of this study are available within the paper.

## Abbreviations

2D: Two dimensional
6MWT: Six minute walk test
ADL: Activities of Daily Living
CI: Confidence Interval
DR: Double row
EnR: Endoscopic repair
FTT: Full tendon tear
Gmax: Gluteus Maximus
GRADE: Grading of Recommendations, Assessment, Development and Evaluation
GTT: Gluteal tendon tear/s
HHS: Harris Hip Score
HOS: Hip outcome score
HRQoL: Hip related quality of life
iHOT: International hip outcome tool
IQR: Interquartile range
JBI: Joanna Briggs Institute
LARS: Ligament augmentation reconstruction system
LEFS: Lower extremity functional scale
MCIC: Minimally clinically important change
MD: Merle d’Aubigne hip score
MF: Microfracture
mHHS: Modified Harris Hip Score
MMT: Manual muscle testing
MRI: Magnetic resonance imaging
NAHS: Non–arthritic hip score
NRS: Numerical Rating scale for pain severity
NSAIDs: Non–steroidal anti–inflammatories
OHS: Oxford hip score
OpR: Open repair
PRFM: Platelet rich fibrin matrix
PRISMA: Preferred reporting items for systematic reviews and meta–analyses
PTT: Partial tendon tear
QoL: Quality of Life
RCT: Randomised controlled trial
SD: Standard deviation
SF: 12/36–12/36–item short form survey
SLS: Single leg stance
SMD: Standardised mean difference
SS: Suture staple
SSS: Sport specific scale
TFL: Tensor fasciae latae
THA: Total hip arthroplasty
TT: Transtendinous
VAS: Visual Analogue scale for pain severity
