# Supplementary material for: Low quality evidence supports surgery for gluteal tendon tears, no non-surgical evidence was identified: a systematic review
Source: BMC Musculoskelet Disord. 2026 Feb 3;27:191. doi: 10.1186/s12891-026-09519-0 (PMC12958556; doi:10.1186/s12891-026-09519-0)
Supplement: Supplementary file 1 — Supplementary Material 1. [file 12891_2026_9519_MOESM1_ESM.docx]

**Appendix 1:**

The search was based on three main concepts: i) people with GTT, ii) treatments undertaken and iii) long-term outcomes

Search term:

i) people with GTT;

(gluteal OR gluteus OR “gluteus minimus” OR “gluteus medius” OR “greater trochanter” OR trochanteric OR “hip abductor” OR “Lateral Hip pain”) AND (pain OR tear OR bursitis OR syndrome OR tendinitis OR tendinosis OR tenosynovitis OR tendinopathy OR avulsion OR rupture OR tear OR GTPS OR “greater trochanteric pain syndrome”)

ii) treatments undertaken;
AND (surgery OR surgical OR arthroscop* OR repair OR endoscop* OR reconstruct* OR suture OR “mini-open” OR “wait and see” OR injection OR physiotherapy OR “physical therapy” OR exercise OR rehabilitat* OR training OR conservative)

iii) long-term outcomes
AND (biomechanic* OR kinetic OR strength OR “quality of life” OR function OR force OR visa-g OR “harris hip score” OR HHS OR “international hip outcome tool” OR iHOT OR outcome* OR Trendelenburg OR VAS OR NAHS OR “non arthritic hip score” OR “Oxford hip score” OR OHS OR “quality of life” OR QoL OR pro OR prom OR “ 12 item short form”)

Total from inception to November 2024

- Cinahl - 951
- Cochrane Library - 114
- Medline -2076
- Scopus - 2865
- Web of Science – 2810
- Clinical Trials.gov – 11

**Examples of studies excluded:**

1. Whiteside LA. Surgical technique: Transfer of the anterior portion of the gluteus maximus muscle for abductor deficiency of the hip. *Clin Orthop Relat Res*. 2012;470(2):503-510. doi:10.1007/s11999-011-1975-y
   *Excluded due to no outcome measures used.*
2. Jimenez-Telleria I, Foruria X, Moreta J, Bernuy L, Casado O, Martinez-de Los Mozos JL. Gluteus Medius Repair using a Gluteus Maximus Flap Augmented with Synthetic Polyethylene Mesh: Surgical Technique and Case Series. *Orthop Rev (Pavia)*. 2022;14(3):31904. Published 2022 Aug 5. doi:10.52965/001c.31904 *Excluded due to less than 10 participants.*
3. Whiteside LA, Roy ME. Incidence and treatment of abductor deficiency during total hip arthroplasty using the posterior approach: repair with direct suture technique and gluteus maximus flap transfer. *Bone Joint J*. 2019;101-B(6_Supple_B):116-122. doi:10.1302/0301-620X.101B6.BJJ-2018-1511.R1

*Excluded due to concomitant procedure, total hip arthroplasty and tendon repair together.*
